# Ferroptosis at the crossroads of epilepsy and neurodegeneration

**DOI:** 10.1097/MS9.0000000000005039

**Published:** 2026-04-28

**Authors:** Sarum Ali Khan, Muhammad Adnan Qamar, Taimoor Ali

**Affiliations:** a Department of Medicine, Wah Medical College, Wah Cantt, Pakistan; b Faculty of Medicine, Spinghar Medical University, Kabul, Afghanistan

**Keywords:** disease-modifying therapy, epilepsy, epileptogenesis, ferroptosis, glutathione, GPX4, iron dysregulation, lipid peroxidation, neurodegeneration, neuronal vulnerability, oxidative stress, reactive oxygen species, regulated cell death, system XC^−^

## Abstract

Ferroptosis, an iron-dependent form of regulated cell death characterized by lipid peroxidation, has emerged as a potential contributor to neuronal injury across neurological disorders. Increasing evidence suggests that ferroptotic mechanisms may participate in the pathophysiology of epilepsy, linking sustained oxidative stress, iron dysregulation, and progressive neuronal loss. Mechanistically, ferroptosis is driven by disruption of the canonical system Xc^−^–glutathione–glutathione peroxidase 4 axis, resulting in impaired detoxification of lipid hydroperoxides and membrane damage accumulation. Neurons are particularly susceptible due to high oxygen consumption, abundant polyunsaturated fatty acids, and vulnerability to iron-catalyzed reactive oxygen species generation. Experimental studies and gene expression analyses have identified ferroptosis-related pathways enriched in epileptic tissue, while human biomarker investigations demonstrate reduced glutathione levels and elevated lipid peroxidation products in patients with epilepsy. Although most therapeutic data derive from preclinical rodent models, pharmacological modulation of ferroptosis – via iron chelation or inhibition of lipid peroxidation – has shown seizure-reducing and neuroprotective effects. Importantly, ferroptosis likely operates alongside other regulated cell death pathways rather than acting as a singular mechanism. Shared features of iron accumulation and lipid peroxidation in neurodegenerative diseases, such as Alzheimer’s and Parkinson’s, further support a broader pathogenic relevance. Continued translational investigation is necessary to determine whether targeting ferroptosis may offer disease-modifying strategies in epilepsy.

*To the Editor,*

The discovery of ferroptosis – an iron-dependent form of regulated cell death characterized by lethal lipid peroxidation – has advanced our understanding of neuronal injury across neurological disorders. Ferroptosis is increasingly recognized as a potential mechanistic contributor in epilepsy, linking sustained oxidative stress, membrane damage, and neuronal loss to broader neurodegenerative processes, while offering novel therapeutic targets. Although previously studied primarily in cancer and acute ischemic injury, its relevance to chronic neurological conditions like epilepsy is emerging.

Epilepsy remains a highly prevalent neurological disorder, with many cases resistant to current therapies. Beyond aberrant neuronal excitability, epilepsy involves persistent oxidative stress, membrane disruption, and progressive neuronal loss – features that have historically lacked a unified mechanistic explanation. Ferroptosis integrates these elements through dysregulated iron metabolism and unchecked lipid peroxidation, both of which are observed in epileptic brain tissue. Iron homeostasis disruption and reactive oxygen species (ROS) generation have been associated with seizure-induced neuronal injury, positioning ferroptosis as a plausible contributor to epileptogenesis^[^1^]^.

Mechanistically, ferroptosis arises from the convergence of excessive lipid peroxidation, glutathione depletion, and dysregulated iron metabolism. The canonical pathway involves inhibition of the cystine/glutamate antiporter (system Xc^−^, encoded by SLC7A11), which impairs cystine uptake, reduces intracellular glutathione synthesis, and impairs glutathione peroxidase 4 (GPX4) activity – the central enzyme that reduces lipid hydroperoxides to non-toxic lipid alcohols. When this defense fails, accumulated lipid hydroperoxides trigger membrane damage. Neurons are particularly vulnerable due to their high oxygen consumption and abundance of polyunsaturated fatty acids (PUFAs), which are prone to peroxidation when antioxidant defenses are overwhelmed. Elevated iron levels catalyze the Fenton reaction, generating hydroxyl radicals that drive membrane lipid peroxidation – a hallmark of ferroptotic cell death^[^2^]^. Gene expression studies have identified ferroptosis-related genes differentially regulated in epilepsy, enriched in oxidative stress and ROS metabolism pathways, suggesting that these processes may actively contribute to neuronal injury and epileptogenic progression rather than being passive bystanders^[^3^]^.

Human biomarker data further support this association. Children with epilepsy exhibit reduced glutathione levels, partial inactivation of GPX4, and elevated 4-hydroxy-2-nonenal (4-HNE), a specific marker of PUFA peroxidation. These findings indicate biochemical overlap between ferroptotic processes and seizure pathology^[^4^]^.

Modulating ferroptosis offers a promising therapeutic avenue. Preclinical studies in rodent epilepsy models demonstrate that pharmacological inhibition – via iron chelators, GPX4 activators, or lipid peroxidation suppressors – can reduce seizure frequency and attenuate neuronal loss. These results shift focus from symptomatic seizure suppression toward enhancing cellular resilience^[^5^]^. However, most evidence remains preclinical; clinical translation faces challenges, including blood–brain barrier penetration, risks of systemic iron depletion, and potential off-target antioxidant effects. No ferroptosis-specific agents have yet entered early-phase clinical trials for epilepsy, highlighting the need for cautious optimism and rigorous translational studies^[^6^]^.

Finally, recognizing ferroptosis as a shared pathway reframes neuronal vulnerability across disorders. Importantly, ferroptosis likely operates alongside other regulated cell death pathways such as apoptosis and necroptosis, rather than functioning as a singular explanatory mechanism. Ferroptosis has been implicated in major neurodegenerative diseases, including Alzheimer’s disease (iron accumulation and lipid peroxidation in amyloid plaques), Parkinson’s disease (dopaminergic neuron loss linked to GPX4 dysfunction), and amyotrophic lateral sclerosis (motor neuron degeneration involving oxidative lipid damage). These parallels suggest that iron dysregulation and lipid peroxidation may represent common drivers, raising the possibility that ferroptosis-targeted therapies could mitigate not only epileptogenesis but also associated cognitive decline or comorbid neurodegeneration^[^7^]^.

In conclusion, ferroptosis provides a compelling mechanistic link between epilepsy and neurodegeneration through iron-driven oxidative damage and lipid peroxidation. Longitudinal human studies, direct validation in epilepsy brain tissue, and careful evaluation of ferroptosis inhibitors are essential next steps to translate these insights into disease-modifying treatments.

## Ethical approval

Not applicable.

## Consent

Not applicable.

## Data Availability

Not applicable.

## Gurantor

Sarum Ali Khan.
